# Early Infirmaries in Methoni During the Venetian Occupation

**DOI:** 10.7759/cureus.54962

**Published:** 2024-02-26

**Authors:** Ioannis A Biris, Andreas I Biris, Spyros N Michaleas, Theodore G Papaioannou, Periklis Panagopoulos, Marianna Karamanou

**Affiliations:** 1 History of Medicine and Medical Ethics, National and Kapodistrian University of Athens, School of Medicine, Athens, GRC; 2 Medicine, University of Nicosia Medical School, Nicosia, CYP; 3 Obstetrics and Gynecology, National and Kapodistrian University of Athens, School of Medicine, Athens, GRC

**Keywords:** medieval messenian colonies, coron, venice, medieval infirmaries, middle ages

## Abstract

Introduction: In the High Middle Ages, Venetian sovereignty was recognized in Methoni and Koroni (in Greece). It helped lay the foundations for the prevalence of democracy in Venice in the Eastern Mediterranean. The development of these two decadent regions of Messinia, had to be supported by social welfare infrastructure. Today, the search for these social welfare infrastructures in Venetian Methoni at the beginning of the conquest turns mainly to the monasteries of the Latin monastic orders that settled there during that time. These spiritual institutions, which provided shelter, hospitality, and even medical care to those in need, have not been identified to date.

Objectives and methods: The goal of this paper is to propose two possible locations based on bibliographic and on-site research. With the help of bibliographies, Venetian files, and relevant wills from the time, an effort was made to identify these ruins. On-field research was carried out to consolidate the findings that arose from the analysis of bibliographic references, the evidence arising from them, as well as local tradition.

Results and conclusion: The monastery of the Cistercian nuns shows that, in the context of charity, medical care was provided to those in need. The monastery in Paliomothoni was one of the earliest infirmaries in Venetian Methoni. The location of the first infirmary of Venetian Methoni within the Cistercian monastery of Paliomothoni is highly probable. Additionally, it was found to be operating there by Dominican monks at a later time.

## Introduction and background

In Paliomothoni, in the region of Methoni (in Greece), are the remains of the foundations of a monastery that can still be found today, a monastery founded by Cistercian nuns at the beginning of the conquest [1,2]. The original monastery in Paliomothoni was under the auspices of the Diocese of Methoni and was dedicated to the Virgin Mary (Santa Maria). It was abandoned and destroyed shortly before 1267, following a revolt by the Greeks in the area [2]. In 1277, Dominican monks settled in the area of Methoni. The traces of the Dominican monastery have not been located inside the castle of Methoni, despite reports to the contrary [3]. Apart from their religious duties, the monks also provided care and shelter for travelers from Venetian ships who were headed to the Holy Land. As these hospitality needs grew, the Dominicans were subsidized for their service by the Venetian Senate in 1323 [3].

Following the expulsion of the Cistercian nuns from the area of Methoni in 1267, sometime later (unidentified to this day), the ruined monastery in Paliomothoni was re-opened, most likely by Dominican monks [3]. This monastery was radically renovated in the following century, and the present-day ruins belong to this second, smaller monastery, which was built on the foundations of the former.

## Review

Methods

The research methodology for the detection of the early infirmaries in Venetian Methoni included the identification of the relevant ruins from the Middle Ages in this area. Subsequently, bibliographies, Venetian files, and relevant wills from the time helped in the effort to identify these ruins. This was followed by field research to consolidate the findings derived from the analysis of bibliographic references, the evidence from these sites, and local traditions.

Results

Following field research, the uncovering of two building phases among the ruins of the monastery in Paliomothoni dating to the Middle Ages proved the initial hypothesis regarding their use. The first and larger monastery of the Cistercian nuns at the beginning of the conquest provided medical care to those in need, whereas the Dominican monks continued providing medical care in the new monastery that was built on the ruins of the former. The latter can be proven by Venetian files that refer to subsidies provided by the Venetian Senate as well as later donations. Thus, the monastery in Paliomothoni is one of the earliest infirmaries in Venetian Methoni.

Discussion

The Venetian presence in Greece and the particular interest of Venice in the area of Messinia date back much earlier than the 10th century. The infiltration of Italian merchants into the Byzantine economy was slow and gradual. Venice, which had been subject to the control of Byzantium until the end of the 10th century, continued as its ally until 1204, receiving significant privileges and rewards at times [4].

The control of Constantinople became a matter of priority for Venice since, in this way, it would certainly prevail in their rivalry with other naval cities in Italy, namely Pisa and especially Genoa [4]. On April 13, 1204, the crusader-pilgrims of the Fourth Crusade captured Constantinople. After the distribution of the conquered Byzantine territories, Methoni and Koroni, which found themselves in significant decline at the time, were soon turned into Venetian naval bases. Following this, the two regions developed significantly in the following centuries and kept growing in strength well until the end of the 15th century [4].

Baudouin de Flandre was given the Latin throne of the Byzantine Empire. Subsequently, the Crusaders divided the profits according to their strength, and, of course, Venice took the lion’s share since its fleet had transported them to Constantinople. Following the distribution of the Romanian territories, the Venetian presence in Messinia consolidated and developed impressively in the following centuries and continued to strengthen until the end of the 15th century [4].

After the occupation of Methoni and Koroni by the Francs in 1205, Venice, to not give up the rights of the territories it had acquired with the distribution agreement (Partitio), sent its fleet to Methoni in 1207. The goal of the Venetians was to consolidate their sovereignty in Methoni and Koroni, the two valuable ports for sea trade and for controlling the roads to the east. However, Methoni had fallen into obscurity. Epidemics and piracy in particular had turned their port into a pirate hideout. The climate of the area at the time is explained by the description of bishop Nikolaos in 1160, and it is revealing of the significant decline: “The city of Methoni, but my renunciation of the lie does not allow me to complete my sentence, was once a city, but now is a desert city, deserted by citizens, deserted by walls and by the security of the walls, but is still called a city nevertheless...” [5].

After the conquest, the Venetian admiral Raniero Dandolo assumed the expenses for the maintenance of the two cities, which were passed to him as fiefs. During the decade between 1220 and 1230, the Serene Republic of Venice granted its possessions to tenants (affictatores), only to later bring them back under its direct control. In 1269, the Senate decided on the fortification of the ruined castle of Koroni and the additional construction of fortification towers in 1283 [6]. In 1292, the construction of the fortress of Methoni was also underway [7].

For the most part, Methoni became a naval base that functioned as a supply station for western pilgrims traveling to the Holy Land but also as a military gathering place for the Venetian naval force in its fight against its rivals as well as for the defense of its eastern colonies from pirates [7]. Almost simultaneously with the conquest of the two Venetian bases in Messinia, monastic orders settled there and established their churches and monasteries inside and outside the castles. The care of the passengers on Venetian ships was essentially left in the hands of these monasteries. A little later, around 1320, Venice adopted a paternalistic attitude towards its citizens and possessions, as the Senate would employ or consent to the employment of physicians or surgeons, who also became officials of the local administration responsible for the care of the sick Venetian officials, officials of the administration, the garrison, and the colonists [7].

**Figure 1:**
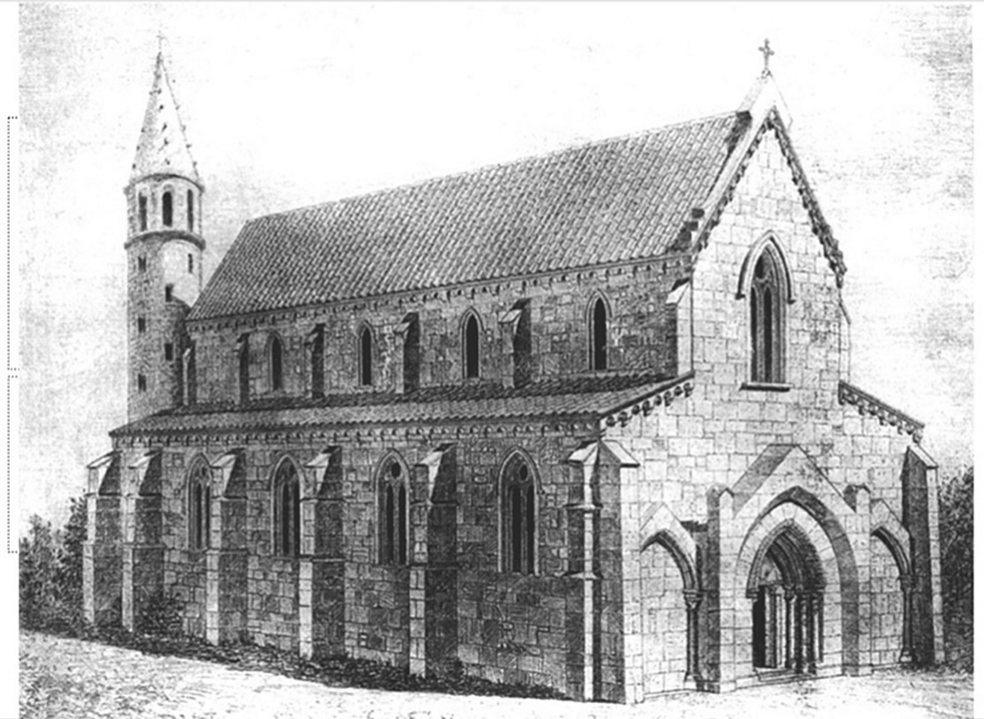
Representation of the Cistercian monastery of Zarakas by Professor Anastasios Orlando licensed under Creative Commons

In Methoni, there was a Latin diocese. Traces of the monastery founded by Cistercian nuns at the beginning of the conquest can still be found today in the surrounding area. A total of 12 monasteries of the order were founded on the Greek peninsula during this period of time [2]. The order had strict principles and rules that prescribed fasting, manual labor, and long periods of silence for its members. The order's facilities were located outside the residential areas, in fertile valleys or fields near water sources. The Cistercian nuns who arrived in Methoni founded the monastery of Santa Maria de Verge (the Virgin Mary) outside the castle, something mentioned in many manuscripts of the time. The monastery is possibly identified with the ruins of the church of Paliomothoni found today, which, after 1930, was wrongly attributed to Agios Leon (*Agioleos*) [3,8]. The monastery in Paliomothoni should not be confused with the church of *Santa Maria alla spiaggia* (Our Lady on the Beach), which was located within the current residential area of Methoni and was destroyed in the 19th century [9]. After the fall of the Latin Byzantine empire and the expulsion of the Westerners from Constantinople, the Greeks revolted and tried to expel them from wherever else they could (Figure 1). Likewise, in Paliomothoni, the nuns were expelled shortly before 1267 and the monastery was abandoned [7].

The ruins of the first phase of the monastery at Paliomothoni bear a striking resemblance to the ruins of Zarakas monastery in Stymphalia, which are preserved in better condition. Both monasteries were built in roughly the same time period by Cistercian monks. Traces of a small infirmary and a pharmacy can be found today among the ruins of the Zarakas monastery. It is highly likely that in Paliomothoni too, despite the current absence of any traces of any healthcare buildings, an infirmary was operating in the surrounding area of the monastery. After all, the largest Cistercian monasteries in Europe had two sanatoriums, one for the monks and one for the laypeople [10]. A careful excavation in Paliomothoni could uncover the location of the infirmary and the other auxiliary spaces of the monastery. At least in the 13th century and until the expulsion of the nuns from Methoni, primary care was provided there as well.

In the area of Methoni, similar activity can be found to have occurred by Dominican monks who arrived in the area before 1277. Apart from their religious duties, they were also active in providing shelter and care to travelers. Given the ever-growing demand for their services, they managed in 1323 to be subsidized by the Venetian senate with two loads of corn for one month a year for four years [11]. The Franciscans of Koroni managed to get a similar subsidy a little later, in 1332 [12].

Medical and hospital services were essential and played an important role in the safety of the Venetian colonies and their ships. It has been reported that Venice supported and developed such services. In the area of the Messinian possessions, the monks had started the operation of the Voldana hospital, which received patients disembarking from the ships. The exact location of this hospital is unknown. Venice, then known as *Commune Veneciarum *(the Commune of Venice) initially refused to subsidize the hospital, but it was eventually subsidized by the governor of Methoni with a free supply of 48 *modii *(an ancient Roman unit of measurement) of wheat from the state stores, something that was deemed illegal [11,12]. The whole reference to the Voldana hospital possibly refers to the already subsidized Dominican monks. With these subsidies from the monastic brotherhoods, Venice covered the accommodation and care needs of travelers in its ports in Messinia, but at the same time gained control and a say in the operation of the sanatoriums and primordial hospitals of the monasteries established there.

The Dominican monastery of Santa Maria in Methoni existed in 1277 and is mentioned again in the lists of the order in 1303 [11]. The two testaments of Franco Polini also confirm the continuous operation of the monastery. The first was drawn up in Methoni on December 9th, 1335 [13]. Within it, Franco Polini chooses the Santa Maria of the Dominicans as his burial place and leaves at the disposal of the executors of his will a sum for his funeral (sacrum passage), along with fifty hyperpyra. Additionally, he left five more hyperpyra to the priest of Saint Johannes of Methoni. However, Polini did not die in the immediate years following 1335. He left a new will drawn up again in Methoni, some 40 years later, on July 2nd, 1375. Within this one, among other things, he reiterated his wish to be buried in Santa Maria of the Dominicans, specifying that he wants to be buried where his father-in-law, Stendal Tiepolo, was buried and that a chapel and an altar be built there [13]. It is fair to assume that the Dominican monastery had been moved outside the castle at some point, before 1335, since a cemetery with a chapel and dedicated altars would require a lot of space and be easier to find outside of the castle’s walls. The hypothesis that the abandoned monastery of the persecuted Cistercian monks in Paliomothoni had become the seat of the Dominican monks also seems probable.

Following the breakout of the plague pandemic in September 1347, the will of the Venetian cleric and canon of Methoni, Marino Soranzo, written on January 12th, 1348, also marks the radical renovation of the monastery of Paliomothoni. Soranzo, while justifying his will, states, among other things, "for this *mortality *that is in the present" [13]. *Mortality *here refers to the plague pandemic, also known as the Black Death.

**Figure 2:**
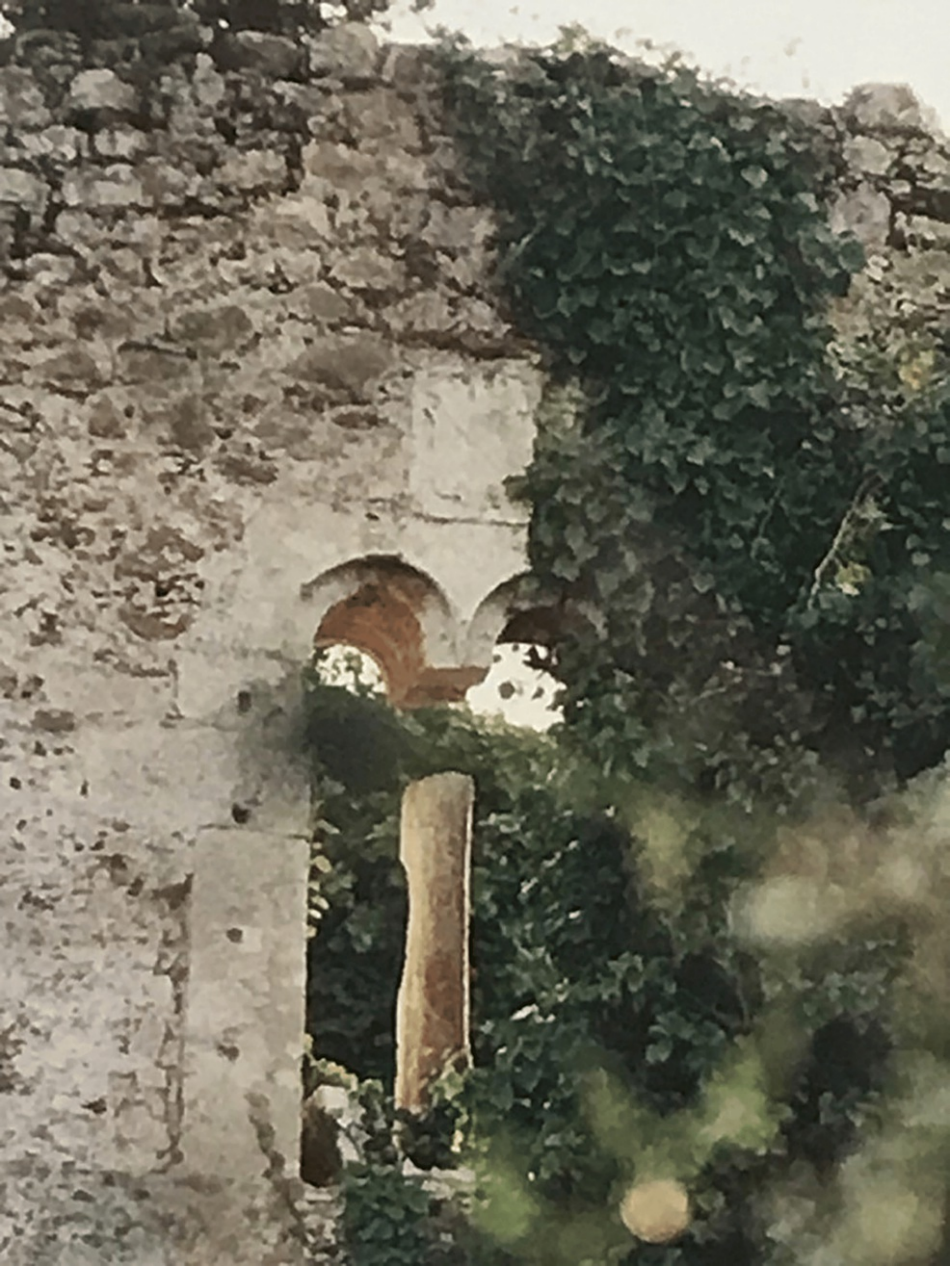
The coat of arms of Soranzo seen above the archway in the monastery of Paliomothoni This photo was taken during field research.

**Figure 3:**
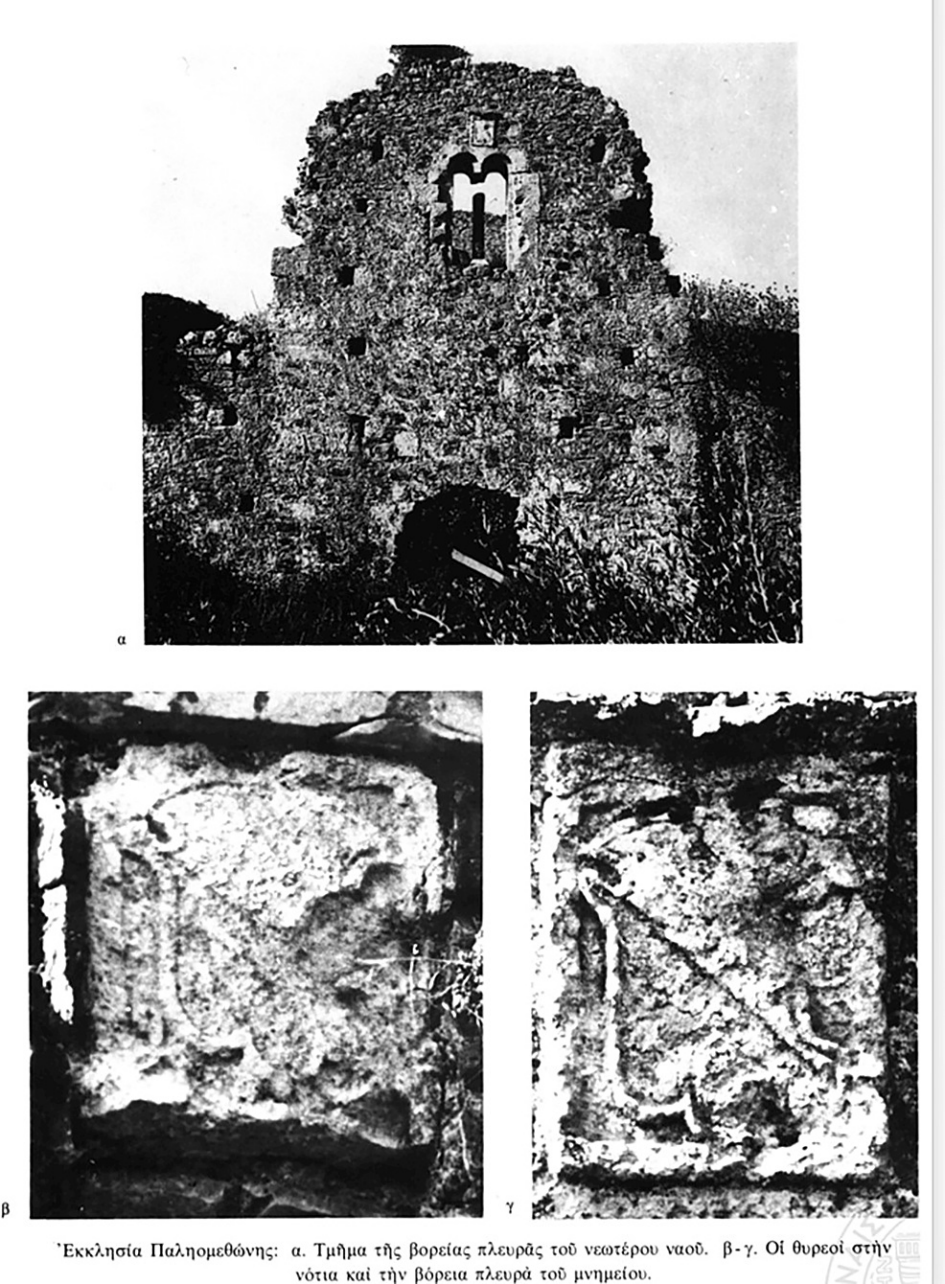
The church of Paliomothoni Source: *Re-examination of the so-called Agioleos near Methoni* (in Greek), by Bouras C. From *Filia Epi Eis Georgion E. Mylonan,* 1989, Athens: The Archaeological Society at Athens.

Soranzo, who managed some fields and half a vineyard of the Dominican monastery on the beach of Methoni, allocated 300 hyperpyra for the purchase of four beds, mattresses, and bedclothes, as well as the proceeds from a part of his property in Methoni for the renovation of the monastery in Paliomothoni and the relief of the poor who arrived there [11]. The renovated monastery was named Santa Maria di Valverde in honor of the new founder and benefactor of the monastery (Marino Soranzo), who was a member of the *Scuola Misericordia*, i.e., the Fraternity of Mercy of Santa Maria di Valverde in Venice [11,14]. It is from this will and the significant donation that the links between the Soranzo and his coats of arms on the building of the second phase of the monastery in Paliomothoni arise (Figures 2-3).

## Conclusions

The location of the first infirmary of Venetian Methoni within the Cistercian monastery of Paliomothoni is highly probable. Additionally, it was found to be operated by Dominican monks at a later time. Surely, after Soranzo's will, the monastery was renovated at the end of 1347 and continued to function well after the plague pandemic, at least until 1375, as shown by the will of Franco Polini. Soranzo's will, with the offer of four beds with mattresses and bedclothes, leaves no doubt about the operation of an infirmary in this region, which was later taken over by the hospital of Saint Johannes in the walled settlement of the Methoni castle, dispensing care to travelers and pilgrims.
